# Detection of viruses associated with bovine respiratory disease complex in samples collected from Albanian cattle during 2022/2023

**DOI:** 10.1099/acmi.0.001053.v3

**Published:** 2026-05-05

**Authors:** Tristan Russell, Majlind Sulçe, Anila Hoda, Xhelil Koleci, Gerald Barry

**Affiliations:** 1 Centre for Experimental Pathogen Host Research, School of Medicine, University College Dublin, Dublin, Ireland; 2 School of Veterinary Medicine, University College Dublin, Dublin, Ireland; 3 Faculty of Veterinary Medicine, The Agricultural University of Tirana, Tirana, Albania; 4 Agricultural University of Tirana, Kodër Kamëz, Tirana, Albania

**Keywords:** Albania, *Betacoronavirus gravedinis*, bovine respiratory disease, *Orthopneumovirus bovis*

## Abstract

The bovine respiratory disease complex causes significant morbidity and mortality with numerous aetiological agents known to contribute to its development, including several bacteria and viruses. In Albania, there is limited information on the prevalence or genetic makeup of viral bovine respiratory disease complex pathogens, which limits mitigation strategies and effective responses such as targeted vaccination against circulating viruses. Nasal or pulmonary samples were collected from cattle in 2022/2023, and then, PCR of bovine *Betacoronavirus gravedinis* (BoCoV) and *Orthopneumovirus bovis* (BRSV) was used to determine their prevalence in Albanian cattle with or without respiratory symptoms. From 105 cattle that were tested, 5 animals tested positive for BoCoV only, 5 animals tested positive for BRSV only and 1 animal tested positive for both BoCoV and BRSV. Four of the eleven positive animals had respiratory symptoms accounting for 8.3% of all symptomatic animals tested. There was an increased prevalence of viruses detected in animals sampled in farms or slaughterhouses within or neighbouring the region of Tiranë compared to other locations. Sequencing of genes encoding the surface proteins enabled phylogenetic analysis and genotyping of Albanian isolates. BoCoV spike and haemagglutinin esterase sequences sat in the European clade, with the spike sequences split across three lineages. BRSV isolates were either in subgroup II, with many other European isolates, or subgroup VIII with several Croatian isolates. This study has shown that BoCoV and BRSV were not highly prevalent in cattle with respiratory symptoms in Albania at the time of sampling. Phylogenetic analyses showed that the detected pathogens are closely related to isolates from other European countries, and there have been multiple introductions of each.

## Data Summary

All sequences used for analysis have been submitted to GenBank and can be accessed using the accession numbers referenced in this manuscript. Sequences obtained from GenBank for phylogenetic analyses are listed in the supplementary information section. Methods for carrying out all analyses are in the Methods section.

## Introduction

The bovine respiratory disease complex (BRDC) encompasses several aetiological and environmental factors that cause respiratory diseases, including tracheitis, bronchitis and pneumonia, in beef and dairy cattle of all ages, though especially feedlot cattle. Symptoms vary on a case-by-case basis with the most common respiratory symptoms, including nasal discharge, dyspnoea and cough, while non-respiratory symptoms include a reduction in milk production and increased body temperature [1]. The most commonly isolated bacteria from cattle with BRDC are *Mannheimia haemolytica*, *Pasteurella multocida*, *Histophilus somni* and *Mycoplasma bovis*, while the most commonly detected viruses include *Varicellovirus bovinealpha1* (formerly *Bovine herpesvirus 1*), *Orthopneumovirus bovis* [also known as bovine respiratory syncytial virus (BRSV)], *Betacoronavirus gravedinis* (BoCoV), *Mastadenovirus bovidae* (formerly bovine adenovirus), *Respirovirus bovis* (formerly bovine parainfluenza virus-3) and *Pestivirus bovis* (formerly bovine viral diarrhoea virus) [2,4]. Non-aetiological factors contributing to BRDC include respiratory dysbiosis, travel-induced stress and genetic variants predisposing cattle to respiratory disease [2,5,8]. Single infections with the above-mentioned pathogens can cause symptoms of BRDC, but normally, co-infections give rise to clinical manifestations, with the most commonly accepted dogma being non-aetiological factors that predispose cattle to more severe viral infections, which create an environment more conducive to secondary bacterial disease [9].

The agricultural sector, especially beef and dairy, makes a significant contribution to the Albanian economy, accounting for 17.7% of gross domestic product in 2021 [10,11], while across the entire European Union, agriculture only contributed 1.3% to gross domestic product in 2023 [12]. In contrast to many European countries, farms in Albania are small with most cattle farmers having less than 11 animals, and the most common breed is the Albanian Shorthorn and crossbreeds, though some other pure breeds have been imported from European countries [10,13]. There is information on the prevalence of some BRDC-associated pathogens in Albania, and private control programmes are implemented on the small number of large commercial dairy farms for *Mycobacterium bovis* and infectious bovine rhinotracheitis (caused by *V. bovinealpha1*), but for most, no active surveillance is conducted [13]. There is almost no information on the genotype or phylogeny of these viruses available on open-source databases. To improve this situation, the prevalence of viruses associated with BRDC in Albania was determined by screening for BoCoV and BRSV in 105 respiratory swabs from cattle with or without a history of respiratory disease. At the time of writing, there are no data on the prevalence of BoCoV or BRSV in Albania [13]. Sequencing of positives enabled phylogenetic analysis and genotyping, which will contribute to the genetic information on viral BRDC pathogens in Albania.

## Methods

All samples were collected as part of routine veterinary work. Ethical approval for analysis of samples was provided by the University College Dublin Animal Research Ethics Committee (AREC-E-22-27-Barry) and the Albanian Ministry of Agriculture and Rural Development, National Veterinary and Plant Protection Authority.

Nasal or pulmonary swabs were collected from 105 cattle with or without symptoms of respiratory disease in Albania during 2022/2023 (Table S1, available in the online Supplementary Material). Five batches of five epidemiologically related nasal swab samples collected from the same region and on the same date were pooled prior to extraction, while 80 samples were not pooled, meaning a total of 85 samples in total. Cattle were assessed by a qualified veterinarian for a history of respiratory disease with postmortem examination of lungs used to assess animals sampled post-slaughter. Lung swabs were collected from the right apical lobe exhibiting visible slight to moderate bronchopneumonia lesions. Swabs were immediately placed in cooler boxes, then transported to the laboratory in 2–12 h, where they were stored at −20 °C until extraction.

Virus was recovered from swabs by placing in 1 ml PBS, vigorously shaking for 5 s and then incubating at room temperature for 5 min with shaking every 1–2 min. The PBS was then used for DNA and RNA extraction (Roche High Pure Viral Nucleic Acid Extraction Kit) following the manufacturer’s protocol. RNA was converted to cDNA using Superscript IV (Thermo Fisher Scientific) following the manufacturer’s protocol. cDNA and the remaining extract were stored at −85 °C until being shipped to University College Dublin School of Veterinary Medicine on dry ice. Samples were screened for BoCoV and BRSV by PCR using primers specific for each pathogen following a published protocol (Table S2) [14]. PCR products were run on 0.75–1.5% agarose gels to identify samples with bands of the expected size (Fig. S1). Sequencing of BoCoV and BRSV genes encoding surface proteins was carried out by PCR amplifying the coding regions; then, PCR products were run on 0.75% agarose gels, and bands of the expected size were purified by gel extraction (TransGen Biotech). Gel extracts were submitted for Sanger sequencing with appropriate forward and reverse primers, and chromatograms were inspected to verify sequence quality (Eurofins Genomics). BoCoV and BRSV sequences were downloaded from GenBank (Table S3), and then, alignments of sequences were carried out using Clustal Omega [15,16] in Jalview software version 2 [17,18]. Phylogenetic trees were generated using the neighbour-joining method [19] in Seaview software version 5 [20] with 100 bootstraps, and the Interactive Tree of Life web server [21] was used to generate tree images.

## Results

**Fig. 1.**
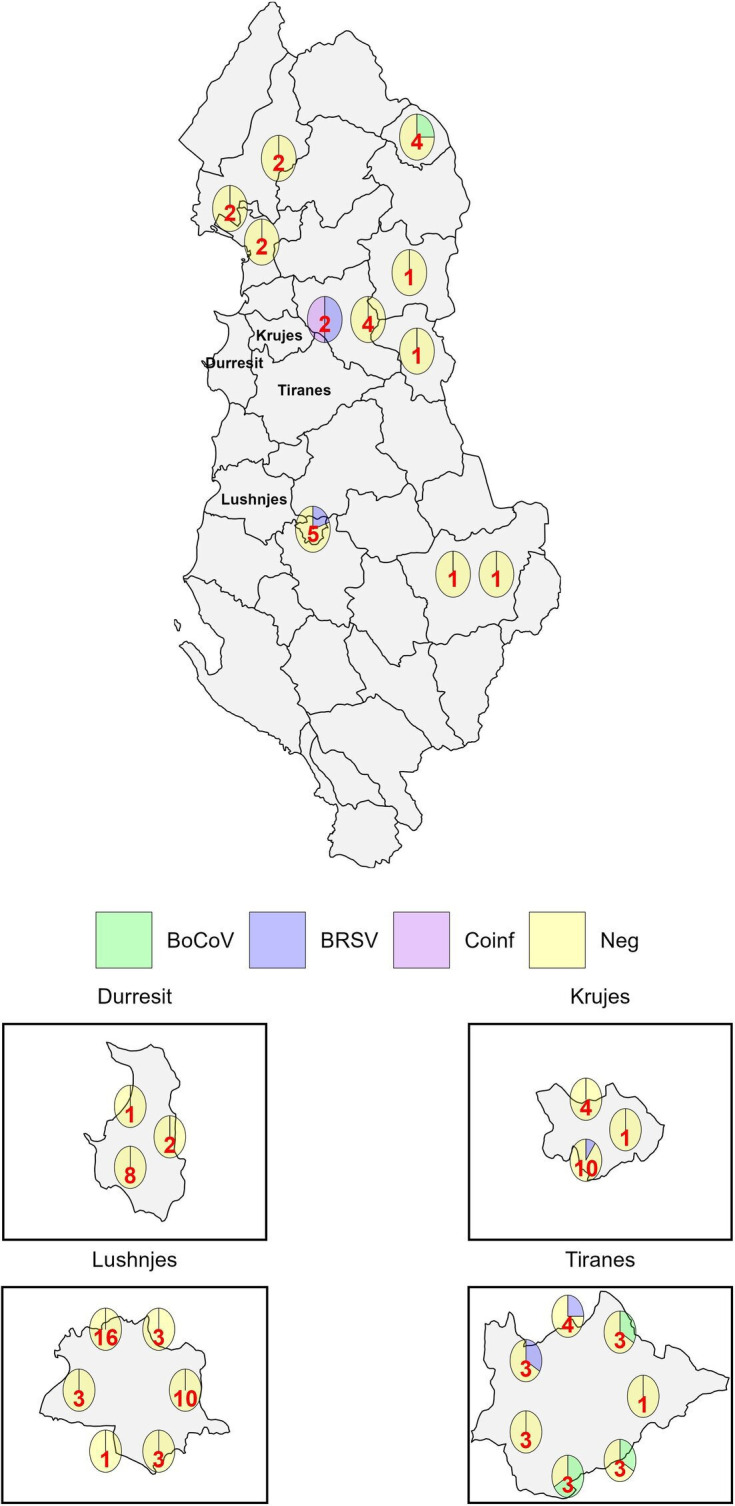
Map of Albania with pie charts showing the surveillance results for each location sampled in a district.The value in red is the number of samples collected from each location.

PCR of 85 respiratory samples, including 5 pools of 5 samples, was used to determine the prevalence of BoCoV and BRSV among cattle in Albania at the time of sampling. BoCoV alone was detected in five samples (4.76%), BRSV alone was detected in five samples (4.76%), and BoCoV and BRSV were detected in one animal (0.95%). None of the pooled samples were positive for BoCoV or BRSV. Two of the six animals positive for BoCoV had respiratory symptoms, and four appeared healthy (Table 1). Five of the BoCoV-positive cattle were calves aged 6–11 months, including the two with respiratory disease, and the other was a 7-year-old adult. The six positive samples were collected from five different locations (Fig. 1) on four different dates, though all had been collected between 24 June 2023 and 1 July 2023 (Table 1). Two of the six cattle positive for BRSV had respiratory symptoms, and four appeared healthy (Table 2). Five were calves between 2.5 months and 11 months old, including one of the two cattle with a history of respiratory disease, while the second was a 12-year-old adult. Positive samples were collected from five locations (Fig. 1) on three different dates with one isolate from February 2023 and the other five from June 2023. The one animal positive for BoCoV and BRSV did not have respiratory symptoms. Six of the eleven animals positive for virus were based in the Tiranë province at the time of sampling, while three of the remaining five animals were in regions that share a border with Tiranë (Fig. 1). Virus detection was more common from asymptomatic animals (18.9%) than animals with respiratory symptoms (8.3%).

**Table 1.** Details of BoCoV-positive samples

Sample no.	Collection date	Location	Symptoms	Swab	GenBank accession no.
3	27 June 2023	Tirana	Respiratory	Nasal	PP156977
9	27 June 2023	Klos	None	Nasal	PP156978
19	27 June 2023	Has	None	Nasal	PP156979
51	01 July 2023	Kamëz, Tiranë	Respiratory*	Lung	PP156980
52	24 June 2023	Kamëz, Tiranë	None	Nasal	PP156981
53	25 June 2023	Paskuqan, Tiranë	None	Nasal	PP156982

*Lung lesions observed postmortem.

**Table 2.** Details of BRSV-positive samples. Sample 62 was not submitted to GenBank due to poor quality of sequence, though it was sufficient to confirm the presence of BRSV

Sample no.	Collection date	Location	Symptoms	Swab	G/F sequence	GenBank accession no.
5	27 June 2023	Laknas, Tirana	Respiratory*	Lung	Partial/full	PP197918
8	27 June 2023	Klos	None	Nasal	None/partial	PP197919
9	27 June 2023	Klos	None	Nasal	Partial/none	PP197920
16	27 June 2023	Tirana	None	Nasal	None/full	PP197921
38	16 February 2023	Tobel, Kukës	None	Nasal	None/full	PP197922
62	05 June 2023	Adriatik, Krujë	Respiratory	Nasal	None/partial	na

*Lung lesions observed postmortem.

**Fig. 2.**
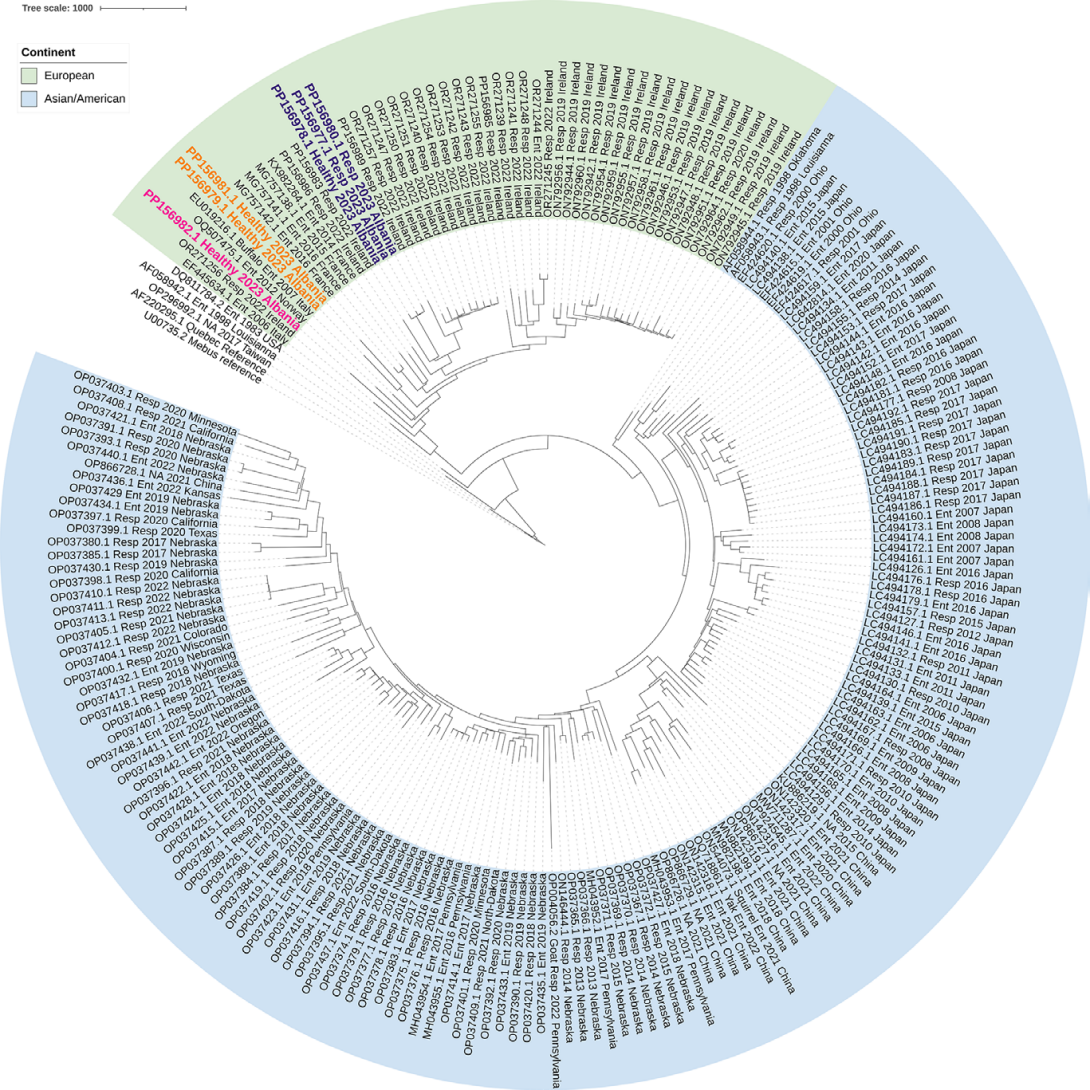
Phylogenetic trees of BoCoV spike HE genetic sequences obtained in this study and those deposited on GenBank. Alignments were made using Clustal Omega, and trees generated using the neighbour-joining method were rooted against the BoCoV Mebus vaccine strain, as this is sufficiently divergent from natural isolates to be an outgroup. Labels for the three subclades of Albanian isolates are shown in pink, orange and blue. The European and Asian/American clades are highlighted in green and blue, respectively.

Sequencing of genes encoding BoCoV and BRSV surface proteins enabled phylogenetic analysis of Albanian isolates. Full-length spike and haemagglutinin esterase (HE) sequences were obtained for the six BoCoV positives (Table 1). Field BoCoV isolates diverge based on location of isolation into two clades on phylogenetic trees. One clade was made up of Asian and American isolates, and a second clade was made up of European isolates. Albanian isolates sat in the European clade of a phylogenetic tree constructed for nucleotide sequences spanning the HE and spike genes (Fig. 2). Sample 52 branched off on its own; samples 19 and 52 sat in their own subclade; and samples 3, 9 and 51 formed their own branch on a subclade with isolates from France and Ireland (Fig. 2).

**Fig. 3.**
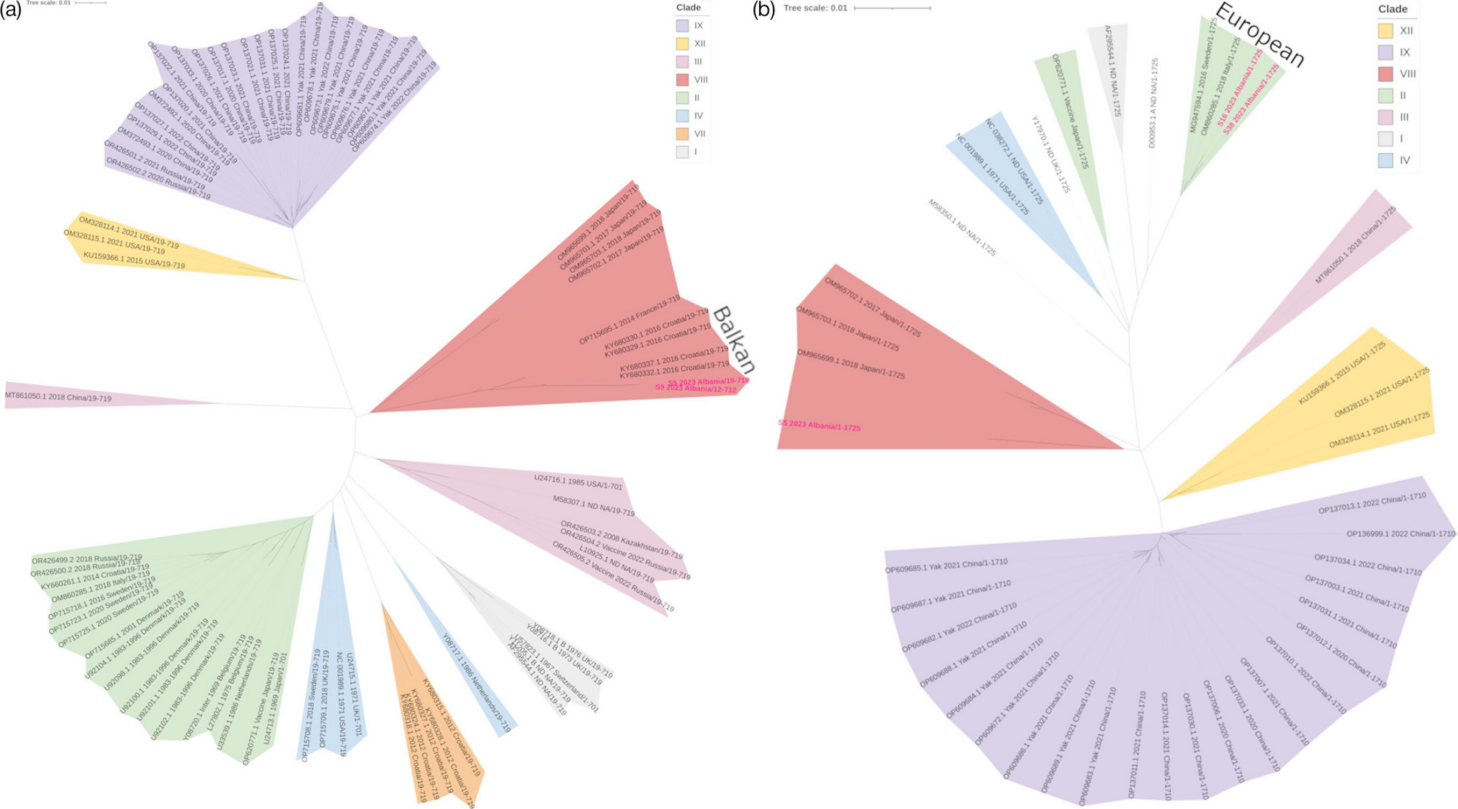
Unrooted phylogenetic tree of BRSV genetic sequences. (**a**) BRSV G gene. (**b**) BRSV F gene. Clades are highlighted with different colours to indicate the different genotype groups. Albanian isolate labels are in pink.

Almost full-length G gene sequences (>90%) were obtained for two BRSV positives, while three full-length and one almost full-length F gene sequences were also obtained (Table 2). The G gene is normally used for genotyping BRSV because it is the most variable, but sequence was only obtained for samples 5 and 9. Phylogenetic analysis of these sequences showed that they were in subgroup VIII, which includes Croatian, French and Japanese isolates (Fig. 3a). Full-length F sequences were obtained for samples 5, 16 and 38. Samples 16 and 38 were closely related sharing a subclade with Swedish and Italian isolates in subgroup II (Fig. 3b). Sample 5, the only isolate included in G and F phylogenetic trees, was on its own branch close to other subgroup VIII isolates, which it grouped with on the G gene phylogenetic tree (Fig. 3b). Sample 8 was closely related to sample 5 when its partial F gene sequence was included (Fig. S2).

## Discussion

A small prevalence study of BoCoV and BRSV was carried out using PCR of nasal or pulmonary swabs obtained from 105 cattle, of which 56.5% had a history of respiratory disease. Overall, virus was detected in 10.48% of all cattle with only BoCoV detected in 4.76% (*N*=5), only BRSV detected in 4.76% (*N*=5) and BoCoV and BRSV detected in 0.95% (*N*=1). One animal showing respiratory symptoms and positive for BRSV was sampled at the slaughterhouse in Tirana district. Non-aetiological factors such as potentially being in the feedlot prior to slaughter, stress associated with travelling to the slaughterhouse and being in an unaccustomed environment could have exacerbated the BRSV infection, and any unidentified BRDC pathogens present, leading to the development of respiratory symptoms. Pulmonary swabs were taken for two of the four virus-positive animals with respiratory symptoms, which accounts for 20% of all pulmonary swabs included in this study. Although a larger sample size is required to draw strong conclusions, these results concur with previous studies showing that lower respiratory tract infections are more often associated with respiratory symptoms [22,24]. Viral pathogens were only detected in 4 of 48 cattle with respiratory symptoms, so there is no evidence that BoCoV or BRSV are major BRDC pathogens in Albania, though the presence of BoCoV and BRSV in asymptomatic cattle means there is potential for these infections to spread in an environment conducive to virus transmission or cause symptoms if the immune response is suppressed or overworked. Serological surveillance of BoCoV and BRSV would provide a more comprehensive assessment of their prevalence by identifying historical infections. Agnostic metagenomic surveillance of respiratory samples would holistically identify the aetiological agents of bovine pneumonia in Albania.

Of the 20 samples collected in Tiranë, 30% were positive for BoCoV or BRSV. Increased detection of BoCoV and BRSV in Tiranë, where there is a more concentrated abundance of cattle farms, suggests that the proximity of animals is driving transmission of viruses. This could be caused by cattle sharing the same land or barns resulting in direct transmission via aerosols or the faecal–oral route. Alternatively, indirect transmission via sharing of equipment or movement of farm workers could be spreading the virus between cattle on nearby farms. Reduced detection of BoCoV and BRSV in more rural farms suggests that geographical isolation reduces virus introduction. There was some relatedness between sequences obtained from isolates collected from nearby locations. BoCoV HE and spike sequences from samples 3, 9 and 51 were similar, and all were collected from within the Tiranë province or a neighbouring province. BRSV-positive samples 5, 8 and 9 were similar with 5 and 9 being on the same subclade in the G gene tree and 5 and 8 being on the same subclade on the partial F gene tree. These sequences were from isolates in or neighbouring Tiranë and were all collected on the same day. There were also exceptions to the geographical influence on isolate relatedness with sequences of some isolates from the Tiranë region not clustering with other isolates from the same area, suggesting that there have been multiple introductions of BoCoV and BRSV into this region.

The BoCoV-positive isolates were all in the European clade of sequences with the divergence across three different subclades, indicating that BoCoV descending from three separate introductions was detected in this study. Albanian BRSV sequences were in subgroups II or VIII, which predominantly contain other European isolates and suggest that BRSV isolates descending from two separate introductions were detected. There have been some imports of dairy cows from Ireland, Italy, Denmark, the Netherlands and Germany [13]. Phylogenetic trees show that Albanian, Irish and Italian BoCoV isolates are related, so introduction of the virus into Albania by cattle imported from Ireland and/or Italy cannot be ruled out. Many Albanian, Danish and Dutch BRSV isolates are in subgroup II showing that they are related, so introduction of BRSV into Albania by cattle imported from Denmark or the Netherlands cannot be ruled out. Similarities between Albanian and Croatian BRSV isolates suggestthere is an intermediate Balkan subgroup within clade VIII, which appears to share a more recent common ancestor than other European isolates. Alternatively, the environment in Balkan States, such as the breed of cattle reared here, or other factors could be shaping the evolution of BRSV in this region.

Failure to obtain good-quality sequence for the G and F genes of the six BRSV-positive isolates is likely due to the high level of variability among BRSV isolates, making the design of universal BRSV primers challenging – partial sequences could be used to design new sequencing primers, but this was not attempted in this study. Whole-genome sequencing will be considered in future studies if the quality of samples allows. Almost full-length G and F sequences were only obtained for sample 5, and it sat in clade VIII for both. Most subgroups annotated on the BRSV trees were previously assigned [4,25,28], but there were some exceptions. Subgroup IX in both BRSV trees was determined due to the similarity of these sequences to Chinese isolates with GenBank accession numbers OM372492.1 and OM372493.1 [4], which have previously been assigned to subgroup IX, though another study also assigned several Brazilian isolates to subgroup IX, and these sequences were not included here [29]. The Chinese and Brazilian isolates may be related, but a phylogenetic tree including both has not been constructed, so it is possible that two separate subgroups will be assigned. Subgroup X, predominantly made up of Japanese isolates, has recently been assigned, but these sequences were not included in this study, so no subgroup X was annotated [28]. An additional subgroup, tentatively assigned XII, was included, as these sequences were uploaded onto GenBank but have not been included in any open-access publications. Subgroup XII contains isolates from the USA that branched off from subgroup IX.

## Conclusions

The prevalence of BoCoV and BRSV during the time of sampling in Albania has been determined. Detection was more common from asymptomatic cattle with only 8.3% of cattle with a history of respiratory symptoms positive for the viral BRDC pathogens included in this study, so there is no evidence to suggest that these viruses were major causes of BRDC in Albania at the time of sampling. Phylogenetic analyses suggested that BoCoV in Albania originates from three separate introductions and BRSV from two separate introductions. Relatedness of the Albanian isolates with other European isolates indicates that the viruses detected in this study descended from cattle imported from another European country. In the case of BRSV, there appears to be a Balkan state intermediate subgroup due to its similarity with Croatian isolates.

## Supplementary material

10.1099/acmi.0.001053.v3 Uncited Supplementary Material 1.

## Abbreviations

BoCoV: *Betacoronavirus gravedinis*
BRDC: bovine respiratory disease complex
BRSV: *Orthopneumovirus bovis*
