# Supplementary material for: Detection of viruses associated with bovine respiratory disease complex in samples collected from Albanian cattle during 2022/2023
Source: Access Microbiol. 2026 May 5;8(5):001053.v3. doi: 10.1099/acmi.0.001053.v3 (PMC13143337; doi:10.1099/acmi.0.001053.v3)
Supplement: Uncited Supplementary Material 1. [file acmi-8-01053-s001.pdf]

Sample No.	Age	Region	Swab	Sampling date	Extraction date	Symptoms and disease
1	13 m	Tirana Farm 1	Nasal	27/06/23	03/07/23	Respiratory
2	12 m	Tirana Farm 1	Nasal	27/06/23	03/07/23	No symptoms
3	11 m	Tirana Farm 1	Nasal	27/06/23	03/07/23	Respiratory
4	10 m	Slaughterhouse, Laknas,Tirana	Pulmonary	27/06/23	03/07/23	No symptoms
5	8 m	Slaughterhouse, Laknas,Tirana	Pulmonary	27/06/23	03/07/23	Respiratory
6	5 m	Slaughterhouse, Laknas,Tirana	Nasal	27/06/23	03/07/23	Respiratory
7	8 m	Slaughterhouse, Laknas,Tirana	Nasal	27/06/23	03/07/23	No symptoms
8	8 m	Klos	Nasal	27/06/23	03/07/23	No symptoms
9	10 m	Klos	Nasal	27/06/23	03/07/23	No symptoms
10	5 m	Mat	Nasal	27/06/23	03/07/23	Respiratory
11	8 m	Mat	Nasal	27/06/23	03/07/23	No symptoms
12	7 m	Mat	Nasal	27/06/23	03/07/23	No symptoms
13	5 m	Mat	Nasal	27/06/23	03/07/23	No symptoms
14	7 m	Tirana Farm 2	Nasal	27/06/23	03/07/23	No symptoms
15	8 m	Tirana Farm 2	Nasal	27/06/23	03/07/23	No symptoms
16	11 m	Tirana Farm 2	Nasal	27/06/23	03/07/23	No symptoms
17	12 m	Has	Nasal	27/06/23	03/07/23	Respiratory
18	5 y	Has	Nasal	27/06/23	03/07/23	No symptoms
19	10 m	Has	Nasal	27/06/23	03/07/23	No symptoms
20	6 y	Has	Nasal	27/06/23	03/07/23	Respiratory
21	5 y	Vajkal, Bulqize	Nasal	21/03/23	04/07/23	No symptoms
22	4 y	Cerma Lushnje	Nasal	02/03/23	04/07/23	Respiratory
23	5 y	Tre urat, Lushnje	Nasal	09/05/23	04/07/23	No symptoms
24	6 y	Tre urat, Lushnje	Nasal	09/05/23	04/07/23	Respiratory
25	10 m	Tre urat, Lushnje	Nasal	09/05/23	04/07/23	Respiratory
26	4 y	Tre urat, Lushnje	Nasal	09/05/23	04/07/23	Respiratory
27	5 m	Tre urat, Lushnje	Nasal	10/05/23	04/07/23	Respiratory
28	4 y	Tre urat, Lushnje	Nasal	11/05/23	04/07/23	Respiratory
29	3 y	Maqellarë, Peshkopi	Nasal	02/03/23	04/07/23	Respiratory
30	4 y	Cerma Lushnje	Nasal	30/05/23	04/07/23	Respiratory
31	2 m	Cerma Lushnje	Nasal	31/05/23	04/07/23	Respiratory
32	6 y	Babrru, Tirana	Nasal	05/04/23	04/07/23	Respiratory
33	5 y	Babrru, Tirana	Nasal	05/04/23	04/07/23	No symptoms
34	4 y	Lushnje (Kacabuni)	Nasal	30/05/23	04/07/23	Respiratory
35	3 y	Libonik, Korçë	Nasal	23/11/22	04/07/23	Respiratory
36	5 y	Tobel, Kukës	Nasal	15/02/23	04/07/23	No symptoms
37	3 y	Tobel, Kukës	Nasal	16/02/23	04/07/23	Respiratory
38	2.5 m	Tobel, Kukës	Nasal	16/02/23	04/07/23	No symptoms
39	2.5 m	Tobel, Kukës	Nasal	17/02/23	04/07/23	Respiratory
40	3 m	Tobel, Kukës	Nasal	18/02/23	04/07/23	Respiratory
41	2 y	Lushnje, Kacabuni	Nasal	06/09/22	07/07/23	Respiratory
42	5 y	Lushnje, Kacabuni	Nasal	06/09/22	07/07/23	No symptoms

43	5 y	Shkodër, H.Brahimi	Nasal	26/07/22	07/07/23	Respiratory
44	5 y	Shkodër, H.Brahimi	Nasal	26/07/22	07/07/23	No symptoms
45	6 y	Balldren, Lezhë	Nasal	11/07/22	07/07/23	Respiratory
46	9 y	Thumanë, Krujë	Nasal	10/11/22	07/07/23	Respiratory
47	5 y	Balldren, Lezhë	Nasal	11/07/22	07/07/23	Respiratory
48	3 y	Lushnje, Farm M.K	Nasal	23/11/22	07/07/23	No symptoms
49	4 y	Drithas, Korçë	Nasal	23/11/22	07/07/23	No symptoms
50	9 m	Kamëz, Tiranë	Pulmonary	01/07/23	07/07/23	Respiratory
51	6 m	Kamëz, Tiranë	Pulmonary	01/07/23	07/07/23	Respiratory
52	10 m	Kamëz, Tiranë	Nasal	24/06/23	07/07/23	No symptoms
53	7 y	Paskuqan, Tiranë	Nasal	25/06/23	07/07/23	No symptoms
54	5 y	Babrru, Tiranë	Nasal	23/06/23	07/07/23	Respiratory
55	7 y	Paskuqan, Tiranë	Nasal	25/06/23	07/07/23	Positive bTB skin test
56	8 y	Paskuqan, Tiranë	Nasal	26/06/23	07/07/23	No symptoms
57	6 y	Lushnje (Farm 1)	Nasal	30/05/23	07/07/23	Respiratory
58	4 y	Lushnje (Farm 1)	Nasal	31/05/23	07/07/23	No symptoms
59	3 y	Lushnje (Farm 1)	Nasal	31/05/23	07/07/23	Respiratory
60	4 y	Tiranë Farm 3	Nasal	04/07/23	07/07/23	No symptoms
61	3 y	Durrës, Katund i Ri	Nasal	03/06/23	08/07/23	No symptoms
62	12 y	Adriatik, Krujë	Nasal	05/06/23	08/07/23	Respiratory
63	9 y	Adriatik, Krujë	Nasal	05/06/23	08/07/23	No symptoms
64	3 y	Borizan, Krujë	Nasal	05/06/23	08/07/23	No symptoms
65	4 y	Slaughterhouse, Tapizë	Nasal	15/06/23	08/07/23	Respiratory
66	10 y	Thumanë, Krujë	Nasal	10/06/23	08/07/23	Respiratory
67	8 y	Thumanë, Krujë	Nasal	10/06/23	08/07/23	No symptoms
68	3 m	Adriatik, Krujë	Nasal	15/06/23	08/07/23	Respiratory
69	9 m	Adriatik, Krujë	Nasal	15/06/23	08/07/23	No symptoms
70	9 m	Adriatik, Krujë	Nasal	15/06/23	08/07/23	No symptoms
71	7 m	Slaughterhouse, Tapizë	Pulmonary	24/06/23	08/07/23	Respiratory
72	7 m	Slaughterhouse, Tapizë	Pulmonary	24/06/23	08/07/23	No symptoms
73	5 m	Slaughterhouse, Tapizë	Pulmonary	24/06/23	08/07/23	Respiratory
74	6 m	Slaughterhouse, Tapizë	Pulmonary	24/06/23	08/07/23	Respiratory
75	11 m	Slaughterhouse, Tapizë	Pulmonary	30/06/23	08/07/23	Respiratory
76	7 m	Durrës, Kënetë	Nasal	30/06/23	08/07/23	Respiratory
77	5 m	Slaughterhouse, Tapizë	Pulmonary	06/07/23	08/07/23	Respiratory
78	5 y	Slaughterhouse, Tapizë	Nasal	06/07/23	08/07/23	Respiratory
79	5 m	Durrës, Kënetë	Nasal	06/07/23	08/07/23	Respiratory
80	5 y	Thumanë, Krujë	Nasal	06/07/23	08/07/23	No symptoms
Pooled samples						
81	3 m	Gorre Lushnje	Nasal	23/07/23	26/07/23	Respiratory
82	2-5 y	Gorre Lushnje	Nasal	23/07/23	26/07/23	Respiratory
83	3-6 y	Tre Urat, Lushnje	Nasal	23/06/23	26/07/23	Respiratory
84	2-6 m	Tre Urat, Lushnje	Nasal	05/07/23	26/07/23	Respiratory

85	3-9 y	Adriatik, Krujë	Nasal	28/06/23	27/07/23	No symptoms

Table S1: *Sample information. Abbreviations: M, month; y, year.*

Gene	Primer sequences (5' – 3')		Annealing (°C)
	Forward	Reverse	
BoCoV primers			
HE	CCCTCATCACCGGCTAGACT	CCCCAAAATTAGCTTCACGAGC	65
HE	CCACTGGATGGGAATTCGTTT	GTAGGTTGTGCAGAGCCATT	63
HE	AGGCTTGTTTTACACTCAGGT	GTACACTTTAAATCTCCTATAACAGC	61
Spike	CTTGGCATTCTTTTGGGTGTTGC	TAATGGAGAGGGCACCGACTT	66
Spike	GGGTTACACCTCTCACTTCT	GCAGGACAAGTGCCTATACC	60
Spike	GTCCGTGTAAATTGGATGGG	TGTAGAGTAATCCACACAGT	60
Spike	TTACAAAAATCAAACACAGACAT	AAACTTTATTACAATCGCTTCC	60
Spike	TCAATTTTTCCCCTGTATTAGG	GTAGTAATAACCACTACCAGTG	59
Spike	TTTAGCTATGTCCCTACTAAGTA	TGTGGTAGCTATTATAATATGCTCG	60
BRSV primers			
G	AGCAACATGCATCAAAGTTAAGC	AGAGAGGATGCCTTGTTGTGG	63
F	AACCCAAAAACAACACGGCA	TGTCACAATACCACCCACGA	64
F	AGACACCCCCTGTTGGAAAC	AAATGCAGGTCTTGGCGGAT	65
Nucleocapsid	GCAATGCTGCAGGACTAGGTATAAT	ACACTGTAATTGATGACCCCATTCT	65

Table S2: *Primer sequences used for PCRs including the annealing temperature used with the High Fidelity Phusion PCR kit (New England Biolabs). Primer pairs used for screening samples are highlighted in yellow. BoCoV primers were taken from this study* [29], *BRSV nucleocapsid primers from this study* [30], *and BHV-1 primers from this study* [31].

BoCoV		BRSV	
Spike	HE	G	F
KX432213.1	MH810163.1	OP137029.1	NC001989.1
FJ415324.1	KX432213.1	OP137027.1	OM860285.1
FJ938065.1	FJ415324.1	OP137026.1	OP620771.1
FJ938066.1	ON093194.1	OP137025.1	OP609672.1
FJ938067.1	DQ915164.2	OP137024.1	OP137034.1
EF424621.1	EU019216.1	OP137023.1	OP137033.1
EF424623.1	AB354579.1	OP137022.1	OP137031.1
FJ425184.1	AF391541.1	OP137021.1	OP137030.1
FJ425185.1	AF391542.1	OP137020.1	OM965703.1
FJ425187.1	KX982264.1	OP137017.1	OM965702.1
FJ425188.1	MG757138.1	OP609681.1	OM965699.1
FJ425189.1	MG757139.1	OP609680.1	OM328115.1
MG518518.1	MG757140.1	OP609679.1	OM328114.1
DQ915164.2	LC494179.1	OP609678.1	MT861050.1
FJ938063.1	LC494180.1	OP609677.1	MG947594.1
DQ811784.2	LC494183.1	OP609676.1	KU159366.1

EF424615.1	LC494184.1	OP609675.1	NC038272.1
EF424616.1	LC494185.1	OP609674.1	NC001803.1
EF424617.1	LC494188.1	OP609673.1	AF295544.1
EU019216.1	LC494189.1	KY680337.1	KM360090.1
AB354579.1	LC494190.1	KY680332.1	OP137014.1
AF391541.1	LC494191.1	KY680330.1	OP137013.1
AF391542.1	LC494126.1	KY680329.1	OP137012.1
KU886219.1	LC494127.1	KY680328.1	OP137011.1
KX982264.1	LC494128.1	KY680327.1	OP137010.1
MG757138.1	LC494129.1	KY680326.1	OP137007.1
MG757139.1	LC494130.1	KY680324.1	OP137006.1
MG757140.1	LC494131.1	KY680318.1	OP137003.1
LC494179.1	LC494132.1	KY680316.1	OP136999.1
LC494180.1	LC494133.1	KY680315.1	OP609689.1
LC494181.1	LC494134.1	KY660261.1	OP609688.1
LC494183.1	LC494135.1	U24716.1	OP609687.1
LC494184.1	LC494138.1	U24715.1	OP609686.1
LC494185.1	LC494139.1	U24713.1	OP609685.1
LC494186.1	LC494140.1	U57823.1	OP609684.1
LC494187.1	LC494141.1	U33539.1	OP609683.1
LC494188.1	LC494142.1	L27802.1	OP609682.1
LC494189.1	LC494143.1	U92104.1	Y17970.1
LC494190.1	LC494144.1	U92102.1	AB245477.1
LC494191.1	LC494147.1	U92101.1	M58350.1
LC494192.1	LC494148.1	U92100.1	D00953.1
LC494126.1	LC494153.1	U92098.1	
LC494127.1	LC494156.1	OM372493.1	
LC494128.1	LC494160.1	OM372492.1	
LC494129.1	LC494161.1	M58307.1	
LC494130.1	LC494162.1	L10925.1	
LC494131.1	LC494163.1	Y08717.1	
LC494132.1	LC494165.1	Y11205.1	
LC494133.1	LC494166.1	Y08718.1	
LC494134.1	LC494167.1	Y08720.1	
LC494135.1	LC494170.1	Y08716.1	
LC494137.1	LC494172.1	OR426505.2	
LC494138.1	LC494173.1	OR426504.2	
LC494139.1	LC494174.1	OR426503.2	
LC494140.1	LC494175.1	OR426502.2	
LC494141.1	LC494177.1	OR426501.2	
LC494142.1	LC494178.1	OR426500.2	
LC494143.1	OP296992.1	OR426499.2	
LC494144.1	AF220295.1	NC_001989.1	
LC494145.1	MH043952.1	OM860285.1	
LC494147.1	MH043953.1	OP620771.1	
LC494148.1	MH043954.1	OP609672.1	

LC494153.1	MH043955.1	OP137033.1	
LC494156.1	LC642814.1	OP137031.1	
LC494157.1	ON792941.1	OP715725.1	
LC494160.1	ON792946.1	OP715723.1	
LC494161.1	ON792947.1	OP715718.1	
LC494162.1	ON792963.1	OP715709.1	
LC494163.1	ON792954.1	OP715708.1	
LC494164.1	ON792956.1	OP715695.1	
LC494165.1	ON792957.1	OP715685.1	
LC494166.1	ON792961.1	OM965703.1	
LC494167.1	U00735.2	OM965702.1	
LC494168.1	AF058943.1	OM965701.1	
LC494169.1	AF058944.1	OM965699.1	
LC494170.1	EF445634.1	OM328115.1	
LC494171.1	AF339836.1	OM328114.1	
LC494172.1	AH014866.2	MT861050.1	
LC494173.1	AH014867.2	MG947594.1	
LC494174.1	AH014868.2	KU159366.1	
LC494175.1	AH014869.2	AF295544.1	
LC494177.1	AH014871.2		
LC494178.1	AH014872.2		
MN982198.1	AH010241.2		
MN982199.1	AH010063.2		
OP296992.1	AH010061.2		
AF220295.1	AH010062.2		
MH043952.1	ON142315.1		
MH043953.1	ON142320.1		
MH043954.1	ON142316.1		
MH043955.1	ON142318.1		
MW711287.1	ON142319.1		
LC642814.1	OP186344.1		
ON146444.1	OP186340.1		
ON792941.1	OP186339.1		
ON792942.1	OP186338.1		
ON792944.1	OP186337.1		
ON792945.1	OP186336.1		
ON792946.1	OP186335.1		
ON792947.1	OP186334.1		
ON792948.1	MH203063.1		
ON792949.1	MH203062.1		
ON792950.1	MH203061.1		
ON792951.1	MH203060.1		
ON792963.1	MH203059.1		
ON792954.1	AF230528.1		
ON792955.1	AF230527.1		
ON792956.1	AF230526.1		

ON792957.1	AF230525.1		
ON792959.1	AF230524.1		
ON792960.1	AF230523.1		
ON792961.1	KM985632.1		
ON792962.1	EU401979.1		
OP037365.1	EU401976.1		
OP037366.1	EU401975.1		
OP037367.1	DQ389650.1		
OP037368.1	DQ389649.1		
OP037370.1	DQ389648.1		
OP037371.1	DQ389647.1		
OP037372.1	DQ389646.1		
OP037373.1	DQ389645.1		
OP037374.1	DQ389643.1		
OP037376.1	DQ389642.1		
OP037379.1	MK903507.1		
OP037380.1	HM573325.1		
OP037381.1	DQ994168.1		
OP037384.1	DQ994167.1		
OP037385.1	MZ825083.1		
OP037386.1	MZ825082.1		
OP037388.1	MZ825079.1		
OP037389.1	MZ825078.1		
OP037390.1	MH475914.1		
OP037391.1	MK095147.1		
OP037392.1	MK095146.1		
OP037393.1	MK095144.1		
OP037394.1	MK095143.1		
OP037395.1	MK095142.1		
OP037396.1	MK095140.1		
OP037397.1	MK095139.1		
OP037399.1	MK095137.1		
OP037400.1			
OP037401.1			
OP037402.1			
OP037403.1			
OP037404.1			
OP037405.1			
OP037406.1			
OP037407.1			
OP037408.1			
OP037409.1			
OP037410.1			
OP037411.1			
OP037412.1			
OP037414.1			

OP037415.1			
OP037416.1			
OP037417.1			
OP037419.1			
OP037420.1			
OP037421.1			
OP037422.1			
OP037423.1			
OP037424.1			
OP037425.1			
OP037426.1			
OP037427.1			
OP037428.1			
OP037429.1			
OP037430.1			
OP037431.1			
OP037432.1			
OP037433.1			
OP037434.1			
OP037435.1			
OP037436.1			
OP037437.1			
OP037438.1			
OP037439.1			
OP037440.1			
OP037441.1			
OP037442.1			
U00735.2			
AF058942.1			
AF058943.1			
AF058944.1			
EF445634.1			
HE616741.1			
HE616739.1			
HE616738.1			
OP186328.1			
OP186327.1			
OP186326.1			
OP186325.1			
OP186324.1			
OP186323.1			
OP186322.1			
OP186321.1			
OP186320.1			
OP186319.1			
OP186318.1			

OP186317.1			
OP186316.1			
OP186315.1			
OP186314.1			
OP186313.1			
MK046011.1			
MK046010.1			
MK046008.1			
MK046007.1			
MK046006.1			
MK046004.1			
MK046003.1			
AY935646.1			
AY935644.1			
AY935643.1			
AY935642.1			
AY935641.1			
AY935639.1			
AY935638.1			
AY935637.1			
KF272919.1			
MW881219.1			
MW881220.1			
OL840236.1			
OL840233.1			
MT975572.1			
MT975571.1			
MT975570.1			
MK903506.1			
MK095186.1			
MK095185.1			
MK095184.1			
MK095183.1			
MK095182.1			
MK095181.1			
MK095179.1			
MK095178.1			
MK095176.1			
MK095175.1			
MK095174.1			
MH203067.1			
MH203066.1			
MH203065.1			
MH203064.1			
MH197039.1			
MH197038.1			

MH197037.1			
KT318124.1			
KT318122.1			
KT318121.1			
KT318120.1			
KT318118.1			
KT318117.1			
KT318116.1			
KT318115.1			
KT318114.1			
KT318113.1			
KT318112.1			
KT318111.1			
KM985631.1			
KF169940.1			
KF169939.1			
KF169938.1			
KF169937.1			
KF169936.1			
KF169935.1			
KF169934.1			
KF169933.1			
KF169932.1			
KF169931.1			
KF169930.1			
KF169929.1			
KF169926.1			
KF169925.1			
KF169924.1			
KF169923.1			
KF169922.1			
KF169921.1			
KF169920.1			
KF169919.1			
KF169918.1			
KF169917.1			
KF169915.1			
KF169914.1			
KF169913.1			
KF169912.1			
KF169910.1			
KF169909.1			
KF169908.1			
EU401989.1			
EU686689.1			
EU401988.1			

EU401987.1			
EU401986.1			
DQ389641.1			
DQ389640.1			
DQ389639.1			
DQ389638.1			
DQ389637.1			
DQ389636.1			
DQ389635.1			
DQ389634.1			
DQ389633.1			
DQ389632.1			
EF193075.1			
OL456213.1			
MW521194.1			
MW521193.1			
MW521192.1			
MW521191.1			
MW521189.1			
MW521188.1			
MW521187.1			
MW521186.1			
MW521185.1			
MW521184.1			
MW521183.1			
MW521182.1			
MW521181.1			
MW521180.1			
MW521179.1			
MW521177.1			
MW521176.1			
MW521175.1			
OL990398.1			
MZ603735.1			
MW711304.1			
MW711303.1			
MW711302.1			
MW711301.1			
MW711300.1			
MW711299.1			
MW711298.1			
MW711297.1			
MW711295.1			
MW711294.1			
MW711293.1			
MW711292.1			

MW711291.1			
MW711290.1			
MW711289.1			
MN982181.1			
MN982180.1			
MN982178.1			
MN982176.1			
MN982175.1			
MN982174.1			
MN982173.1			
MN982171.1			
MN982170.1			
MN982169.1			
MN982167.1			
MN982166.1			
MG757144.1			
MG757143.1			
M64668.1			
M64667.1			
HM573330.1			
HM573326.1			
DQ389660.1			
DQ389659.1			
DQ389658.1			
DQ389657.1			
DQ389656.1			
DQ389655.1			
DQ389654.1			
DQ389653.1			
DQ389652.1			
D00731.1			
EU814647.1			
EU814648.1			
MH741424.1			

Table S3: *GenBank accession numbers for all sequences used in phylogenetic analyses.*

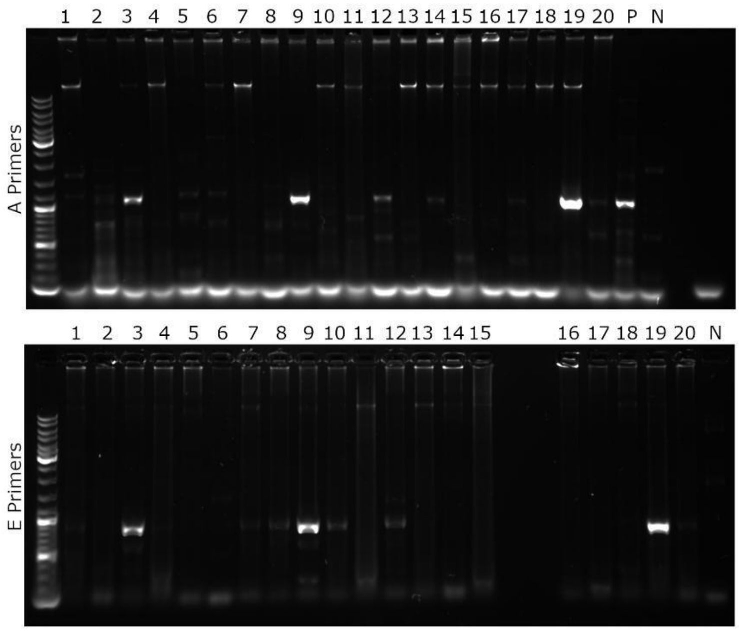

Supplementary Figure S1: *Representative DNA agarose gels for primer pairs A and E used to identify BoCoV positive samples.*

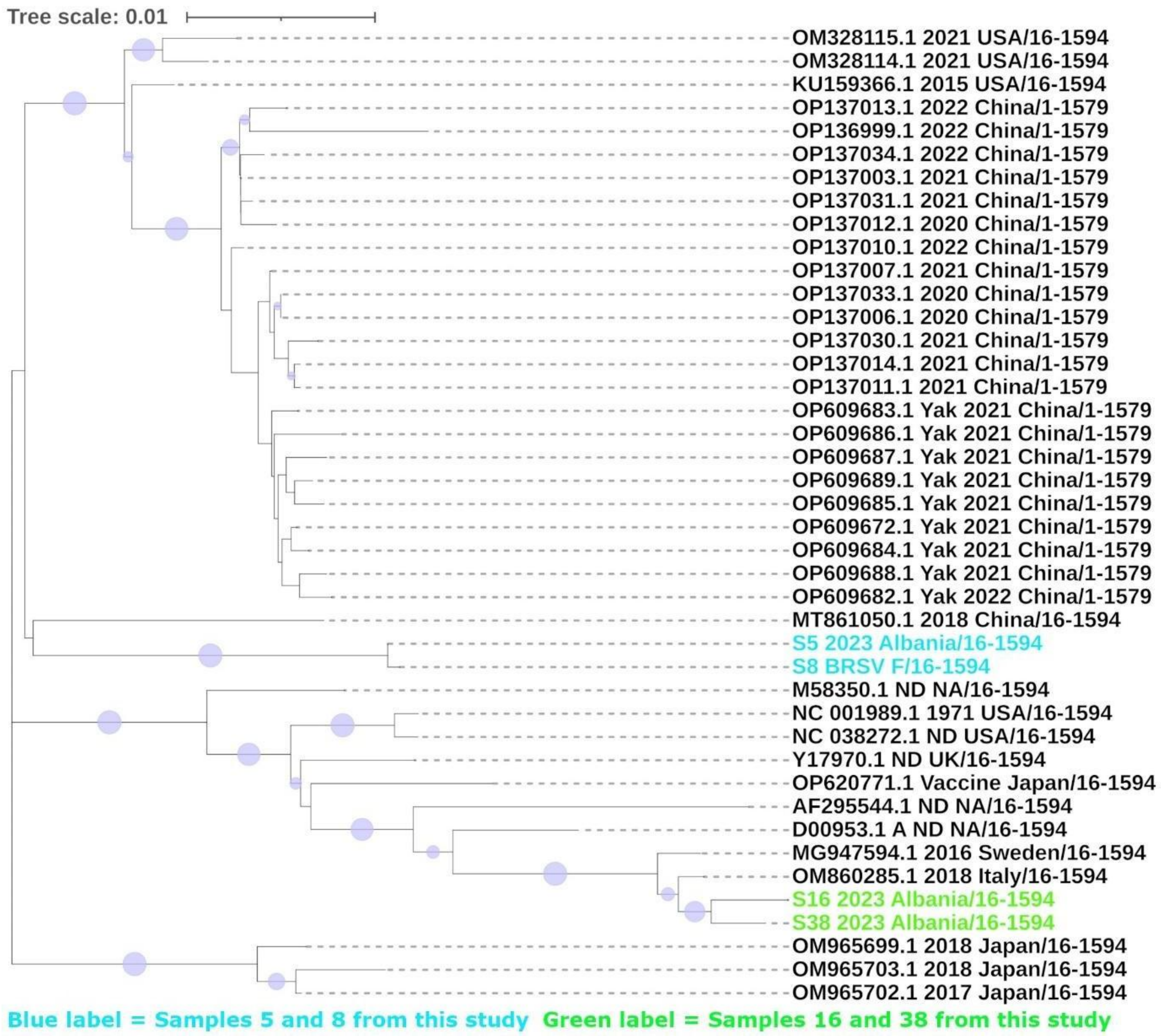

<u>Figure S2:</u> *Unrooted phylogenetic tree of partial BRSV F nucleotide sequences. Sequences of the alignment were trimmed to cover only the partial sequence of Sample 8. Coloured labels indicate the closely related Albanian isolates from this study. Circles represent branch support from 100 bootstraps with larger circles indicating stronger support.*
